# Motion dazzle and camouflage as distinct anti-predator defenses

**DOI:** 10.1186/1741-7007-9-81

**Published:** 2011-11-25

**Authors:** Martin Stevens, W Tom L Searle, Jenny E Seymour, Kate LA Marshall, Graeme D Ruxton

**Affiliations:** 1 Department of Zoology, University of Cambridge, Downing Street, Cambridge CB2 3EJ, UK; 2 College of Medical, Veterinary & Life Sciences, University of Glasgow, Glasgow G12 8QQ, UK

## Abstract

**Background:**

Camouflage patterns that hinder detection and/or recognition by antagonists are widely studied in both human and animal contexts. Patterns of contrasting stripes that purportedly degrade an observer's ability to judge the speed and direction of moving prey ('motion dazzle') are, however, rarely investigated. This is despite motion dazzle having been fundamental to the appearance of warships in both world wars and often postulated as the selective agent leading to repeated patterns on many animals (such as zebra and many fish, snake, and invertebrate species). Such patterns often appear conspicuous, suggesting that protection while moving by motion dazzle might impair camouflage when stationary. However, the relationship between motion dazzle and camouflage is unclear because disruptive camouflage relies on high-contrast markings. In this study, we used a computer game with human subjects detecting and capturing either moving or stationary targets with different patterns, in order to provide the first empirical exploration of the interaction of these two protective coloration mechanisms.

**Results:**

Moving targets with stripes were caught significantly less often and missed more often than targets with camouflage patterns. However, when stationary, targets with camouflage markings were captured less often and caused more false detections than those with striped patterns, which were readily detected.

**Conclusions:**

Our study provides the clearest evidence to date that some patterns inhibit the capture of moving targets, but that camouflage and motion dazzle are not complementary strategies. Therefore, the specific coloration that evolves in animals will depend on how the life history and ontogeny of each species influence the trade-off between the costs and benefits of motion dazzle and camouflage.

## Background

Across the animal kingdom, risk of predation has led to the evolution of anti-predator defenses. Of these, defensive coloration (including camouflage, startle displays, warning signals, and mimicry) is widespread [1-3]. Recently, the study of camouflage in particular has seen a resurgence across a wide range of disciplines [3]. Recent experiments have shown that disruptive coloration, involving high-contrast markings that break up the body outline and shape, is an effective method of concealment over and above simply matching the background [4-8]. However, despite recent significant advances, two major gaps in our understanding of camouflage exist.

First, it remains controversial whether some animal markings can inhibit the predator's judgment of the speed and trajectory of a moving prey animal and thus inhibit capture, a phenomenon termed 'motion dazzle' [9,10]. Such markings are thought to include the high-contrast bands, stripes, and zig-zag markings common in snakes, fish, mammals (for example, zebra), and various invertebrates (for example, fast-flying butterflies). To date, only three studies have investigated motion dazzle. Our previous study [11] used a computer game, in which human subjects had to capture moving prey with different markings. This study found that while an unpatterned target matching the background luminance was hardest to capture, targets with certain striped and zig-zag markings made capture more difficult compared with uniform conspicuous targets. However, although these results are suggestive, the study used a limited number of striped patterns, and did not compare these with other arrangements, such as camouflage markings. Thus, it remains unclear whether patterning itself can interfere with motion perception, or whether specific marking arrangements are needed for this. A second experiment [12] investigated markings expressed by the cuttlefish, *Sepia officinalis*, when stationary and when moving. They found that cuttlefish adopted low-contrast markings when moving, implying that high-contrast patterns might actually facilitate capture. Third, a recent psychophysical experiment [13] investigated how different types of markings (similar to those used in our previous study [11]) could affect speed perception in human participants who had to choose which of two stimuli moved more quickly. The researchers found evidence that markings can affect speed perception, but unlike in [11], they did not have an unmarked camouflage control with which to compare the markings. In addition, their study was designed to test potential effects of dazzle coloration in human conflict, such as a combatant throwing a grenade at a moving vehicle, rather than in nature. Despite the above limitations, these studies provide initial evidence that motion dazzle could work, and high-contrast dazzle markings were once common during wartime on ships, mainly in an attempt to hinder targeting by enemy submarines [14]. In addition, analyses of snake markings indicate an association between marking type and movement patterns [15-17], implying that dazzle coloration may exist in nature.

Second, we currently have little understanding of how different types of anti-predator coloration relate to each other [9]. Theory predicts that disruptive coloration should be most effective when comprising high-contrast markings [1,18]. This prediction is supported by some recent work [4], provided that pattern contrast does not exceed that found in the background [5,8]. High contrast is also often believed to be used in motion dazzle, potentially affording a dual benefit for some pattern types, being effective for both motion dazzle and disruptive camouflage [18]. However, human psychophysical experiments indicate that low contrast makes objects appear to move more slowly [19-22], causing underestimates of motion. In some cases, low-contrast gratings appear to move up to 50% more slowly than high-contrast equivalents moving at the same speed [21]. To date, no experiments have tested the role of contrast in motion dazzle or in a prey-capture style task.

In this paper, we present experiments using human subjects attempting to capture artificial computer 'prey' (as in our previous study [11]) with different markings and contrast. We tested capture success (number of targets caught and the number of missed attempts) using prey with dazzle coloration (stripes), camouflage markings that were either background matching (where the markings were a random sample of the background, but with the stipulation that no markings touched the body edge, as in [4]), or disruptive coloration (where the markings were a sample of the background with at least some pattern components touching the body edge [4]), and unpatterned targets (camouflage gray and conspicuous white). Experiments were conducted on both moving and stationary targets. We tested (i) if some specific pattern arrangements inhibit successful capture when moving, (ii) the role of contrast in motion dazzle, and (iii) whether some pattern types could offer dual protection, that is, camouflage when stationary, and of motion dazzle when moving.

## Results

### The role of pattern in motion dazzle

**Figure 1:**
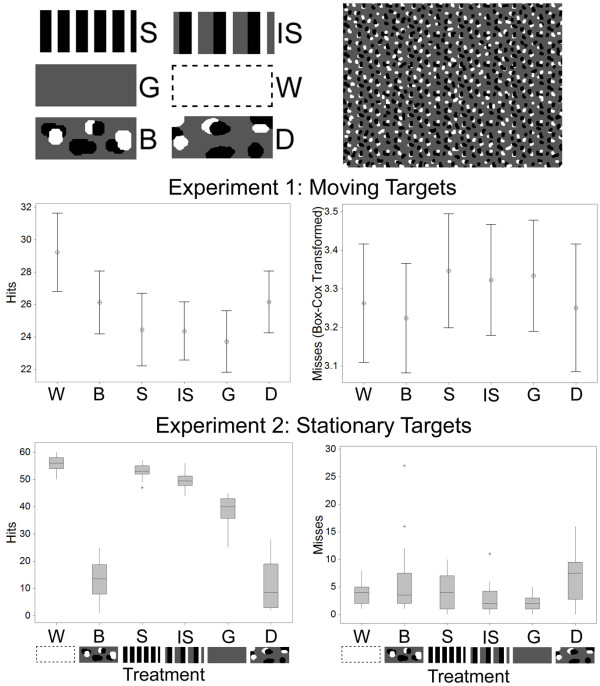
**Stimuli used in experiments 1 and 2, and results**. Top panels: stimuli and an example background used in experiment 1 and 2. Target types had: black and white stripes (S), interval stripes of gray, white, and black (IS), uniform camouflage gray (G), uniform conspicuous white (W), background matching (B), and disruptive (D) coloration (see main text for details). All target types except W have the same average luminance as the background. Middle and lower panels: the mean number (plus 95% confidence interval (CI)) of successful captures (left panels) and missed attempts (right panels) of the different target types in experiment 1 (moving targets), and medians (plus interquartile range (IQR)) for experiment 2 (stationary targets).

Experiment 1 tested how capture success of moving targets was influenced by pattern type (Figure 1; see Methods). Based on previous work [11], we predicted that uniform gray (G), striped (S), and interval-striped (IS) targets would be difficult to capture, and that the uniform white (W) would be captured with relative ease. In addition, we also explored how background matching (B) and disruptive camouflage (D) patterns affected capture success (see Figure 1 for images of the different target types).

For all experiments, we recorded the number of prey items of a given type captured per minute ('hits'), and the number of times that a subject attempted to capture a moving target but missed in their attack and touched the background part of the screen instead ('misses'; see Methods). Participants were presented with six one-minute trials, one for each prey type (with presentation order balanced across all subjects; see Methods).

For experiment 1, capture success (hits) data showed that there was a significant effect of treatment (that is, of prey type: *F*_(5, 359) _= 10.53, *P *< 0.001; see Figure 1). We used planned contrasts whenever possible in our analysis because these are more powerful than using a series of unplanned *post hoc *comparisons or simply comparing confidence intervals. These showed that the conspicuous uniform white target was caught more often than the other treatments (*F*_(1) _= 38.43, *P *< 0.001), and the uniform gray target was caught less often than the aggregate of the patterned prey (*F*_(1) _= 13.12, *P *< 0.001). Targets with stripes were caught less often than the camouflage (disruptive and background matching) targets (*F*_(1) _= 8.37, *P *= 0.004). There was no difference in capture success between the banded and interval-striped targets (*F*_(1) _= 0.01, *P *= 0.928), or between the background matching and disruptive targets (*F*_(1) _< 0.001, *P *= 0.967).

Unlike our previous study [11], we were also able to record how many unsuccessful (missed) capture attempts each subject made for a given prey type. There was a non-significant trend for a difference between the treatments (*F*_(5, 359) _= 2.06, *P *= 0.070). Planned comparisons showed that the uniform gray targets were missed more often than the patterned target types (*F*_(1) _= 4.22, *P *= 0.042), and the targets with stripes were missed more often than the camouflaged targets (*F*_(1) _= 8.05, *P *= 0.005). There was no significant difference in misses between the white target and all other prey types (*F*_(1) _= 0.71, *P *= 0.399), between the disruptive and background matching targets (*F*_(1) _= 0.26, *P *= 0.610), or between the prey with interval stripes and those with banding (*F*_(1) _= 0.24, *P *= 0.627) (Figure 1). Therefore, overall, the uniform gray and dazzle targets were caught less often and missed more often than the other target types.

### The role of pattern in preventing detection and capture when stationary

Experiment 2 tested the ability of participants to detect (and therefore capture) stationary prey of the same treatments used in experiment 1. In this experiment (and experiment 4) capture success (hits) corresponded to when a subject successfully located and touched the target. The misses (or false detections) data corresponded to when a person misidentified part of the screen as a target when the target was in fact elsewhere, and consequently they touched the wrong location on the screen. Therefore, in the stationary experiments, the results correspond more to signal-detection theory, where subjects can correctly detect and attack a target; not detect a target; or erroneously 'detect' a target and attack part of the background. We predicted that the camouflage (background matching and disruptive) targets would have low capture rates, whereas the conspicuous white targets would be detected easily. The uniform gray target might have intermediate protection, because it matched the overall luminance of the background, but lacked the background patterns. The regular high-contrast patterns of the striped prey might either make them conspicuous, or act in disruptive camouflage.

There was a significant effect of treatment on capture (detection) success (S_(5) _= 86.87, *P *< 0.001; Figure 1). The white target was caught more often than the banded target (S_(1) _= 16.00, *P *< 0.001), the banded target was caught more often than the target with interval stripes (S_(1) _= 14.22, *P *< 0.001), the interval-striped target was caught more often than the uniform gray target (S_(1) _= 18.00, *P *< 0.001), and the gray target was caught more often than the background matching target (S_(1) _= 18.00, *P *< 0.001). There was no difference between the background matching and disruptive targets (S_(1) _< 0.01, *P *= 1.000).

There was a significant difference between the treatments for the number of times targets were missed (S_(5) _= 17.16, *P *= 0.004; Figure 1). The gray target was missed less often than each of the disruptive (S_(1) _= 9.00, *P *= 0.003), background matching (S_(1) _= 7.12, *P *= 0.008), and white targets (S_(1) _= 6.25, *P *= 0.012). There were non-significant trends (using sequential Bonferroni critical *P*-value threshold adjustment; see Methods) between the banded and gray targets (S_(1) _= 4.57, *P *= 0.033), and between the white and interval-striped targets (S_(1) _= 3.56, *P *= 0.059). The low number of misses of the gray compared with the camouflaged targets was probably because patches of the background were unlikely to be misidentified as this target type because of its uniform block of color. The relatively high number of misses for the white target may be because it was so easy to detect that subjects struck quickly towards it and sometimes missed. However, without latency-to-strike or other similar data, this is speculative. Overall, when stationary, the camouflaged targets were caught least often and caused most false identifications, whereas the white and striped targets were caught most often.

### The role of pattern contrast in motion dazzle

**Figure 2:**
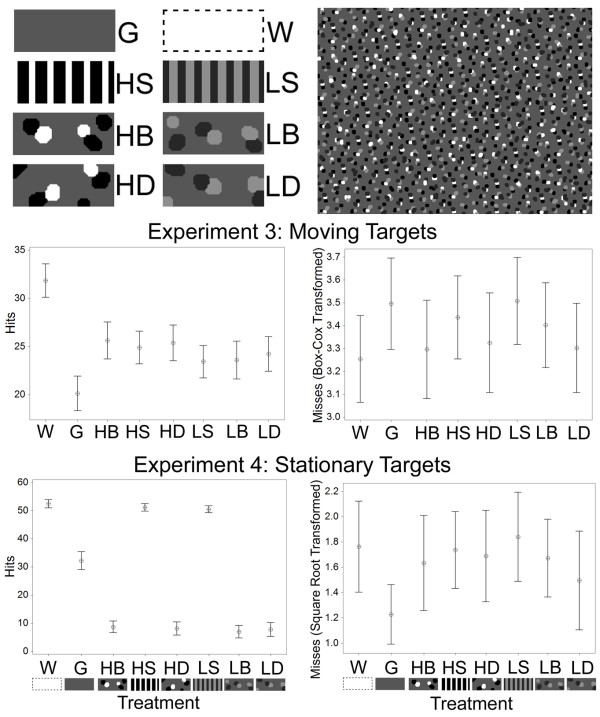
**Stimuli used in experiments 3 and 4 and results**. Top panels: stimuli and an example background used in experiments 3 and 4. Target types are: uniform camouflage gray (G), uniform conspicuous white (W), high-contrast black and white striped (HS), low-contrast gray striped (LS), high-contrast background matching (HB), low-contrast background matching (LB), high-contrast disruptive (HD), and low-contrast disruptive (LD) patterns (see main text for details). All target types except W have the same average luminance as the background. Middle and lower panels: the mean number (plus 95% confidence interval (CI)) of successful captures (left panels) and missed attempts (right panels) of the different target types in experiment 3 (moving targets) and experiment 4 (stationary targets).

Experiment 3 tested the effect of pattern contrast on motion dazzle. In addition to the uniform gray (G) and white (W) targets used in experiments 1 and 2, we used targets that had striped (banded) markings of either high contrast (black and white stripes; HS) or low contrast (intermediate gray stripes; LS) and camouflage markings of high or low contrast that were either disruptive (HD and LD) or background matching (HB and LB) patterns (see Figure 2 for images of the stimuli). Based on previous work, we predicted that motion dazzle would be more effective with low-contrast markings (see above).

For capture success there was a significant effect of treatment (*F*_(7, 511) _= 39.76, *P *< 0.001; Figure 2). The white target was caught more often than all other target types (*F*_(1) _= 174.48, *P *< 0.001), and the patterned targets were caught more often than the uniform gray target (*F*_(1) _= 58.55, *P *< 0.001). The low-contrast patterned targets were caught less often than the targets with high-contrast markings (*F*_(1) _= 14.71, *P *< 0.001). Specifically, the low-contrast striped target was caught less often than the high-contrast striped target (*F*_(1) _= 5.59, *P *= 0.022), and the low-contrast camouflaged targets were caught less often than the high-contrast camouflage targets (*F*_(1) _= 11.06, *P *= 0.001). There was no difference in capture success for the background matching and disruptive targets (*F*_(1) _= 0.14, *P *= 0.704), or between the banded and camouflaged prey (*F*_(1) _= 1.56, *P *= 0.213).

For the number of missed attempts, there was also a significant effect of treatment (*F*_(7, 511) _= 7.09, *P *< 0.001). The white target was missed less often than the other target types (*F*_(1) _= 12.40, *P *< 0.001), and the patterned targets were missed less often than the uniform gray target (*F*_(1) _= 8.51, *P *= 0.004). In addition, the striped targets were missed more often than the camouflaged targets (*F*_(1) _= 20.17, *P *< 0.001). There were non-significant trends for the low-contrast targets to be missed more often than the high-contrast targets (*F*_(1) _= 2.92, *P *= 0.088), including for the low-contrast striped target being missed more often than the high-contrast equivalent (*F*_(1) _= 2.87, *P *= 0.096). There were no significant differences between the low- and high-contrast camouflaged targets (*F*_(1) _= 1.27, *P *= 0.261), or between the disruptive and background matching prey (*F*_(1) _= 0.92, *P *= 0.339). Therefore, the gray target was captured less often than the other targets, and the gray and striped targets were missed more often than the other target types. As predicted, targets with high-contrast patterns were caught more often and missed less often than those of low contrast.

### The role of contrast in concealment

The final experiment tested the relative advantages of contrast and pattern type on the same treatments as used in experiment 3, but when stationary. We predicted that the camouflaged targets would be captured (detected) less often than the uniform targets, and that the uniform gray target should have a lower capture risk than the white and striped targets. Based on previous work [4], disruptive targets might be more effectively concealed when of high rather than low contrast.

For capture success, there was a significant effect of treatment (*F*_(7, 191) _= 537.28, *P *< 0.001). The white target was caught more often than striped target types (*F*_(1) _= 24.63, *P *< 0.001), and the striped targets were caught more often than the gray target (*F*_(1) _= 340.54, *P *< 0.001). The gray target was captured more often than the camouflaged targets (*F*_(1) _= 428.15, *P *< 0.001). There was no difference in capture risk between the disruptive and background matching targets (*F*_(1) _= 0.01, *P *= 0.906), between the high and low-contrast targets overall (*F*_(1) _= 0.05, *P *= 0.817), between the high and low-contrast stripes [*F*_(1) _= 2.84, *P *= 0.112], or between the high and low-contrast disruptive targets (*F*_(1) _= 0.07, *P *= 0.789).

For misses (false detections), there was a significant effect of treatment (*F*_(7, 191)1 _= 2.12, *P *= 0.045). The gray target was missed less often than the camouflaged targets (*F*_(1) _= 7.40, *P *= 0.008) and less often than the striped targets (*F*_(1) _= 16.08, *P *< 0.001). Otherwise, there were no significant differences between the white and striped targets (*F*_(1) _= 0.04, *P *= 0.834), the disruptive and background matching targets (*F*_(1) _= 0.25, *P *= 0.617), the targets with high- or low-contrast patterns (*F*_(1) _= 0.02, *P *= 0.879), those with high- versus low-contrast striped (*F*_(1) _= 0.57, *P *= 0.460), or those with high- and low-contrast disruptive patterns (*F*_(1) _= 1.76, *P *= 0.203). Overall, these results show that the camouflaged targets were captured least often, and the white and dazzle targets captured most often. There was no effect of contrast on either captures or misses.

## Discussion

In this study, we found that moving prey with striped markings were captured less often than targets with either camouflage patterns or uniform conspicuous white targets. Therefore, specific pattern arrangements do promote motion dazzle, making the speed and trajectory of moving targets difficult to judge accurately. Although in experiment 3 there was no difference in mean capture rate between the camouflaged and striped targets, the striped prey were missed significantly more often than the camouflaged targets. This indicates that participants found striped prey more difficult to capture, and made more misdirected attacks. Thus, in both experiments 1 and 3, there was clear evidence that striped targets induce motion dazzle. However, as in our previous study [11], the unpatterned gray target was at least equally as effective as the striped prey at reducing capture. In experiment 3, the reduced effectiveness of stripes compared with the gray target may be because the background was more complex than in experiment 1, comprising five shades of gray instead of three. Previous work shows that background texture may affect speed perception [23], and the effectiveness of motion dazzle may be partly background-dependent. In stationary prey, background complexity can also increase the detection times of camouflaged prey [24].

Targets were more effective at preventing capture when of low contrast. This is consistent with studies of cuttlefish markings [12] and with human experiments indicating that low contrast can cause underestimation of speed [19,21,22]. Dazzle markings, therefore, may be most effective when of low contrast. In addition, high-contrast patches and edges might present positional cues to detect and track motion [25]. In our study, all stimuli were achromatic. However, future work should investigate motion dazzle in chromatic stimuli, both because many animal markings have high chromatic contrast, and because at slow speeds and with low-contrast stimuli, speed discrimination is worse for chromatic compared with achromatic stimuli, and perceived stimulus speed can show a greater dependence on chromatic contrast than on luminance [20,26]. In addition, our set-up only recorded whether a person missed or successfully captured a target, but not whether subjects were drawn to attack and miss the trailing edge, for example. This would be valuable to explore in future work.

While our experiments show that motion dazzle can effectively prevent capture, the mechanisms underlying how motion dazzle works are unclear, although a range of possibilities exist [11,27]. One possibility relates to the so-called 'aperture problem', where the motion of a line viewed through a narrow window is ambiguous for motion parallel to the line itself, and only movement perpendicular to the line is detectable. If movement is detected by local receptive fields that are combined to produce a global estimate of motion, then the true movement of a striped object may be difficult to judge [27]. In addition, the advantage of striped patterns may stem from the repeating nature of the markings, as spatial frequency can affect speed perception [28], and such markings may fatigue or cause adaptation in motion-sensitive cells [29]. In contrast, blotches and spots may provide reference points to facilitate effective tracking and may underlie the ineffectiveness of camouflage patterns in motion dazzle.

In the experiments in which targets were stationary, those with camouflaged markings were captured least often. By contrast, the striped prey was caught often (only the conspicuous white targets were captured more frequently). Thus, although striped markings are highly effective in reducing capture when moving, they are costly when stationary. By contrast, camouflage patterns are ineffective at reducing capture during movement, but provide strong protection when stationary. Our study indicates that those markings that are effective in motion dazzle are not effective in camouflage, and *vice versa*. This is consistent with evidence showing that many animals, including insect larvae and snakes, change their color patterns during development and growth [30,31]. However, markings used in motion dazzle might still have other functions. For example, the zig-zag markings on some snakes seem to function as distinctive warning signals rather than as disruptive camouflage [32,33]. Furthermore, high contrast or conspicuousness may not always be a key aspect of effective warning signals, but rather of communicating distinctiveness from profitable prey [34,35]. Thus, a dual benefit of motion dazzle and aposematism may not arise as a result of high contrast, but rather of distinctive pattern arrangements. In addition, some striped patterns, such as the blue and yellow markings on marine fish, can have a distance-dependent function, acting as a means of communication in close proximity, but camouflage from a distance [36]. Once camouflage is broken, motion dazzle may allow the prey animal to escape.

Our results provide evidence that prey can obtain protection from predators through using aspects of their appearance confusing predators' estimation of prey trajectory (motion dazzle), and that such protection can come at a cost of reduction in crypsis when the prey is stationary. Our results also suggest that the range of appearance types capable of showing such effects may extend beyond the repeated contrasting stripes previously considered. Specifically, we found that uniform gray prey had similar properties to striped prey with regard to protection when moving but vulnerability when still. This has potential implications both for our expectation of the importance of motion dazzle in the natural world, and for understanding the sensory and cognitive mechanisms that might underlie it. It would be useful to explore the generality of this result with respect to background complexity, prey shape and movement behaviors. We also note, as above, that there may be a range of selection pressures on prey appearance (for example, warning signals, sexual selection), making uniform coloration in nature relatively uncommon. Our results show that when animals are under selection for patterned appearance, certain arrangements of markings can be especially important in motion dazzle.

We found no difference between background matching or disruptive patterns for prey, either when moving or stationary. This may have been because the background and prey were comprised of discrete blotches, rather than of more continuous patches of color, brightness and pattern, under which circumstances clear survival advantages of disruptive prey have been shown previously [4,5,7,8]). By contrast, recent aviary experiments using birds foraging for artificial prey against similar background types to those used here also failed to find a survival advantage of disruptive targets over background matching prey [24]. In both that study and the present one, there was no advantage of high contrast for disruptive prey. Therefore, the survival advantage of disruptive coloration may often be background-dependent.

In this study, the effect size for appearance on predation risk of moving prey was generally less than the analogous effect size for stationary prey. It is well known that crypsis is a highly effective mechanism to avoid predation. However, crypsis can impose various restrictions and opportunity costs on animals, including being limited to one or a few background types, and restrictions on movement that is known to facilitate detection of cryptic prey that would otherwise be hard to detect when stationary. This means that for many species, crypsis is not an effective strategy. Animals that are bright and conspicuous, such as those with visual warning or sexual signals, often cannot rely on crypsis. In such cases, the benefits of motion dazzle may be greatest. In addition, the effect of motion dazzle may be substantially enhanced if prey respond to detecting a predator by fleeing; even if they do not move around much ordinarily, motion dazzle may still be important if predators generally see them in motion rather than when stationary. However, a full evaluation of the relative selection pressures on crypsis and motion dazzle requires consideration of how frequently prey are in motion when in close proximity to hunting predators. This is likely to be quite variable between species, and even between sexes or age classes within a species.

Our aim in this paper has been to provide a first test of the hypothesis of conflict between motion dazzle while a target is moving and crypsis when that target is stationary. We have achieved such a 'proof of concept', and found that the potential for such conflict does exist. As such, we consider that further work into this question is justified. We see two distinct but connected routes to further progress. First, it is now important to explore the biological importance of the trade-off between motion dazzle and concealment. We used human participants in this study for convenience, for ethical reasons, and because of the considerable effect that the concept of dazzle has had on naval camouflage. A first step to exploring generality across species might involve a similar experimental set-up to that used here but with non-human predators: birds can readily be trained to peck at targets on a computer screen and this has been used effectively to explore questions in adaptive coloration [37]. Interestingly, Bain *et al*. [38] showed that humans and pigeons produced very similar rankings of the accuracy of different hoverflies' mimicry of wasps, suggesting that cognitive systems related to prey detection and recognition may be similar across phylogeny. Tests involving predator behavior closer to real predation events, using physical models of prey with different appearance, should also be possible. As an example, Powell [39] used captive birds of prey attacking moving weasel models to explore the adaptive function of tail coloration; a similar system could be used to explore the issues considered here, perhaps using snake models. In real predator-prey systems, it should also be possible to obtain reliable data on prey-movement speeds and banding properties (spatial frequency and contrast of stripes), and reasonable information is known about many key predators (such as birds) and their temporal vision, potentially allowing modeling of prey patterns and movement in terms of visual mechanisms.

Second, now that we have shown that conflict can occur, it would be valuable to adopt existing methods in experimental psychology to explore the underlying mechanisms in terms of predator sensory and cognitive systems. Our experiments were not in the form of a 'pure' psychophysical experimental design, but rather were intended to reflect a more natural prey-capture task. Both approaches have their merits, and more conventional psychophysical experiments will be useful to test the ideas and results presented here. Our results for the trials with moving targets are consistent with those of other recent psychophysical work investigating military-style dazzle camouflage, in which humans were presented with a binary choice task involving stimuli of two pattern types, and asked to specify which of the two stimuli appeared to move more quickly [13]. It would be useful for future studies to measure eye movements for human subjects given similar tasks to those considered here, but in a more controlled environment, in order to explore how different types of stimuli and different backgrounds influence visual search and object tracking.

## Conclusions

The idea that animals can combine multiple types of protective coloration is widespread, but empirical investigation is lacking. Our study shows that animals may face trade-offs between the type of color patterns they have, and the functions of those markings. It may often not be possible to combine multiple functions with the same pattern, and the likelihood of this may be strongly dependent upon the circumstances. In general, the defensive strategy that animals use may be strongly linked to their life history, developmental stage, and habitat. For example, highly mobile and active animals found in open environments may benefit more from motion-dazzle markings, whereas animals that rest during the day and are found on a limited number of backgrounds may do best by being camouflaged.

## Methods

We produced a computer game created using Scratch software (2009, version 1.4; http://scratch.mit.edu/), in which human participants attempted to find and capture targets against a background. The general design of the study followed a recent experiment [11], except that we used a touchscreen rather than asking participants to capture targets with a mouse and cursor. This change, with participants directly touching the screen to capture prey, made the task more realistic. In experiments 1 and 3, a single 'prey' item (target) moved at a constant speed against the background: 25 cm/s (approximately 31.13° visual angle per second). In addition to average speed, the distance of target displacement between consecutive refreshes of the screen as it moves may also be important in influencing the outcomes of motion-detection mechanisms, as outlined in previous work with humans [40]. Our display refreshed at 75 Hz, which would equate to a frame-to-frame displacement of 0.4° visual angle (see below). However, we calculated that the software used refreshed at approximately 40 Hz, which would result in a frame-by-frame displacement of about 0.77 degrees. This relatively large value may make our findings regarding dazzle effects conservative, as past work indicates that human motion detection may work most effectively at short displacements. Therefore, it is possible that smaller displacements (produced by faster screen refresh rates) would result in greater motion-dazzle effects than those reported here.

In the game, the prey changed direction unpredictably between 1° and 3° clockwise during movement, and bounced back from the edges of the background with the addition of a 45° anticlockwise turn (that is, the 45° change was in addition to the normal effect of bouncing back off the screen edge, to make the target trajectory less predictable). After successful capture the prey disappeared, and after a delay of 0.5 seconds, reappeared in a random position on the screen. The patterns on the prey rotated as the main body of the target rotated. For example, the striped patterns were always perpendicular to the direction of movement, irrespective of which direction the target was actually travelling.

In experiments 2 and 4 prey were stationary, and the task was to find and then capture a prey item. Once a target was caught, a uniform gray background appeared for 0.5 seconds, before the experimental background reappeared with a new prey target in a random location. This was essential because if the prey item simply reappeared in a new location after capture, its reappearance would reveal its new location. For all experiments, within a single trial, participants had to capture as many prey targets of the same type as possible within one minute. This was repeated for each type of prey target (treatment). Treatment order was balanced in all experiments so that each treatment appeared an equal number of times in each order. We recorded the number of prey items captured, where a subject managed to touch the point on the screen at which the moving prey item was located at that time. We also recorded the number of missed attempts. Missed attempts in the moving-prey trials were the times when subjects saw the target but misdirected their attacks and touched the background part of the screen instead of the prey item. In the stationary prey trials, the subjects incorrectly touched the screen where they thought the target was located, but it was in fact elsewhere (false detection). Note that we did not expect that misses and hits should be inversely related, because several factors can affect this relationship; for example, a subject may concentrate more on capturing a target that seems to be moving faster, and make fewer capture attempts (and thus also fewer misses).

Prey targets and backgrounds were achromatic (shades of gray) created using Photoshop Elements (version 7.0; Adobe Systems Inc., East Oldsmar, FL, USA) as high-resolution, low-compression JPEG files. In all experiments, targets were 2 cm wide and 0.9 cm tall (3.11° and 1.12° visual angle subtended on the viewer's eye). Targets were presented against patterned artificial backgrounds comprising black, white and gray markings on a uniform gray. Several versions of the backgrounds were used in each experiment (six in experiments 1 and 2, and eight in experiments 3 and 4; for all backgrounds used see Additional file 1) to remove any potential interactions that could occur between a given treatment and a specific background arrangement. All experiments were conducted on the same 15 inch (38 cm) touchscreen monitor (Elo 1515L; Tyco Electronics, Shanghai, China) with a refresh rate of 75 Hz (higher than the 60 Hz from our previous study [11]). The flicker of the striped targets was 62.5 Hz (based on calculating the time taken for one complete cycle of white and black stripes), and although relatively high, is still lower than the refresh rate of the display. We calibrated the visual contrast of the different shades of gray displayed on the prey and background in terms of luminance (cd m^-2^), using a luminance meter (Minolta LS-110; Osaka, Japan), as described previously [11]. We determined the background value that would correspond to an intermediate level of gray between black and white, and for experiments 3 and 4, to several intermediate shades of gray. The luminance values were as follows (in cd m^-2^): white = 196, black = 8, gray = 40, light gray = 90, and dark gray = 18. The contrast values, based on Michelson contrast, were 0.66 for white/black against intermediate gray, and 0.38 for light gray/dark gray versus intermediate gray. All treatment types, except the white target, had the same average luminance as the background, and so the key difference between them was in their patterning and contrast. Participants were positioned in front of the touchscreen, approximately 46 cm away, under ambient light conditions (standard fluorescent office lights) kept approximately constant.

In all experiments, participants (166 in total: 60 in experiment 1, 18 in experiment 2, 64 in experiment 3, and 24 in experiment 4) were volunteers naïve to the experimental aims, and were predominantly undergraduate students with normal vision or corrected-to-normal vision. We gave participants only the information needed to undertake the trials. No subject participated more often than once across all experiments. All participants carried out a one-minute practice trial before the main experiment, in which they had to capture a uniform black prey item against a white background. Neither this background or prey target was used in any of the main experiments.

### Statistical analyses

The statistical approach has been described previously [11]. Where possible, we analyzed the results with general linear models (GLMs), with the factors of prey type and order of presentation, with the subject as a random factor. When the data violated the assumptions of a GLM and could not be successfully transformed, we analyzed the results with a non-parametric Friedman test. For most experiments we used planned *post hoc *comparisons [41] by rerunning the main test with the factor prey type replaced with each comparison in turn, using no more comparisons than spare degrees of freedom. This is a much more powerful approach than undertaking multiple unplanned comparisons, and best reflects our specific hypotheses [11,41]. In experiment 2, for the data on number of misses, planned comparisons were not intuitive, and so we reran the main test with all pairwise comparisons, and used a sequential Bonferroni procedure to adjust critical *P*-value thresholds to control for multiple testing. We did not always use the same comparisons for the data on hits and misses because these data can reveal different aspects of the subject's strategy and success, and need not be inversely related (see above and Discussion).

### Experiment 1: The role of pattern arrangement in motion dazzle

We used six prey types: a uniform gray (G) target matching the average background luminance; a conspicuous uniform white (W) target; a target with perpendicular black (3 mm wide) and white (2 mm wide) alternating stripes (S); a target with pairs of 3 mm black and 1.2 mm white stripes separated by interval gray stripes of 4 mm (IS); a camouflaged background matching target (B), consisting of a random sample of the background with the stipulation that no markings touched the target edge; and a camouflaged disruptive prey type (D), comprising samples of the background with at least some patterns located on the target edge (Figure 1).

The results were analyzed with a GLM. We included the uniform white prey item in the overall treatment test because there was no prior reason to believe that this should be treated differently from the other conspicuously-marked treatments. In any case, we focused specifically on planned comparisons between individual prey types rather than on omnibus comparisons across all treatments in our interpretation of results. For the data on misses, we had to transform the data to the power of 0.06, calculated using a Box-Cox procedure (we found a Box-Cox transformation produced the most effective transformation in this case). For our planned comparisons, we compared (i) white versus the aggregate of all other prey types, (ii) gray versus the aggregate of all patterned prey types, (iii) dazzle prey (stripes and interval stripes) versus the camouflage prey (background matching and disruptive coloration), (iv) striped versus interval stripes, and (v) background matching versus disruptive coloration.

### Experiment 2: The role of pattern arrangement in preventing detection and capture when stationary

Experiment 2 comprised the same treatments as in experiment 1, but the targets were stationary. Our aim was to test whether, when stationary, the camouflaged-prey targets would be harder to detect (and therefore capture) than either the dazzle or uniform targets.

The main results and *post hoc *comparisons for both captures and misses were analyzed using a Friedman test. For the capture data, we predicted that the conspicuous white target would be the easiest to capture, and the background matching and disruptive targets most difficult. In addition, although their average luminance matched the background, the arrangement of the striped patterns might render the striped and interval-striped prey more conspicuous than the uniform gray prey. Alternatively, the stripes may function in disruptive camouflage. Based on these predictions, we conducted a series of planned stepwise comparisons, comparing treatments with successively lower capture levels in turn. This resulted in comparisons of (i) W versus S, (ii) S versus IS, (iii) IS versus G, (iv) G versus B, and (v) B versus D. For the misses it was difficult to make clear *a priori *predictions. Therefore, we compared all treatments with each other, and then ranked contrasts in terms of *P *value (lowest to highest). We then selected the comparisons with the five smallest *P *values, and used critical *P*-value adjustment based on a sequential Bonferroni correction.

### Experiment 3: The role of pattern contrast in motion dazzle

In experiment 3, there were eight treatments: a uniform white (W) target; a uniform gray (G) target; background matching targets with either low-contrast (LB) light and dark gray spots, or high-contrast (HB) with black and white spots; disruptive targets of either high (HD) or low (LD) contrast; and striped prey with markings of either high (HS) or low (LS) contrast. In ensuring both types of patterned prey had the same average luminance, we slightly modified the width of the stripes in the low-contrast striped prey (2 mm light-gray and 2 mm dark gray stripes). The backgrounds comprised an average intermediate gray, plus white, black, light-gray, and dark-gray spots in approximately equal proportion.

Results for both captures and misses were analyzed with GLMs. Planned comparisons were: (i) white versus the aggregate of all prey types, (ii) gray versus the aggregate of all patterned prey types, (iii) the aggregate of the low-contrast patterned prey versus the aggregate of the high-contrast prey, (iv) low-contrast stripes versus high-contrast stripes, (v) low-contrast camouflaged disruptive and background matching versus high-contrast camouflaged prey, (vi) striped versus camouflage prey, and (vii) background matching versus disruptive.

### Experiment 4: The role of contrast in concealment

The design of experiment 4 and treatments followed that of experiment 3, but with stationary targets. Results were analyzed with GLMs, with data for the number of misses being square root transformed. There was a single prominent outlier in this data even after transformation. However, rerunning the analysis with this outlier excluded did not change the results (the effect of prey type became more significant without the outlier included). We conducted the following planned comparisons: (i) white versus the striped prey, (ii) stripes versus the gray target, (iii) gray versus the camouflage targets, (iv) high versus low-contrast prey, (v) disruptive versus background matching, (vi) high versus low-contrast stripes, and (vii) high versus low-contrast disruptive prey.

## List of abbreviations

B: background matching pattern; D: disruptive-camouflage pattern; G: uniform gray; GLM: general linear model; HB: high-contrast background matching pattern; HD: high-contrast disruptive pattern; HS: high-contrast striped pattern; IS: interval-striped pattern; LB: low-contrast background matching pattern; LD: low-contrast disruptive pattern; LS: low-contrast striped pattern; W: uniform white.

## Authors' contributions

All authors designed the experiments; MS, WTLS, JES, and KLAM undertook the experiments; MS and GDR performed the statistics, and MS, GDR and KLAM wrote the manuscript. All authors read and approved the final manuscript. The authors declare that they have no competing interests.

## Supplementary Material

Additional file 1 **Additional backgrounds used in the experiments**. Images of the different background samples used in the experiments. Click here for file
