# Supplementary material for: Motion dazzle and camouflage as distinct anti-predator defenses
Source: BMC Biol. 2011 Nov 25;9:81. doi: 10.1186/1741-7007-9-81 (PMC3257203; doi:10.1186/1741-7007-9-81)
Supplement: Additional file 1 — Additional backgrounds used in the experiments. Images of the different background samples used in the experiments. [file 1741-7007-9-81-S1.DOC]

**Additional File 1: Motion Dazzle and Camouflage as Distinct Anti-Predator Defenses**

Martin Stevens, W. Tom L. Searle, Jenny E. Seymour, Kate LA Marshall & Graeme D. Ruxton


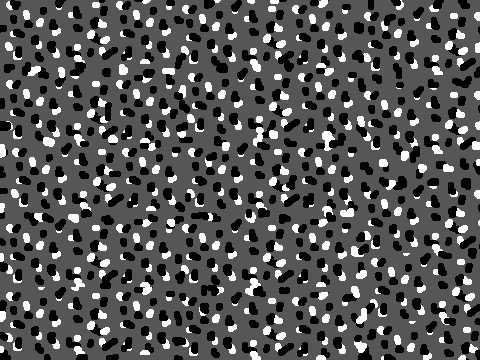

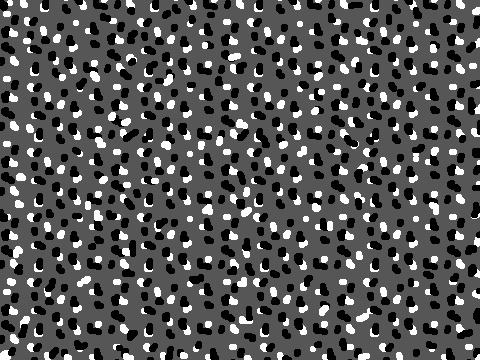

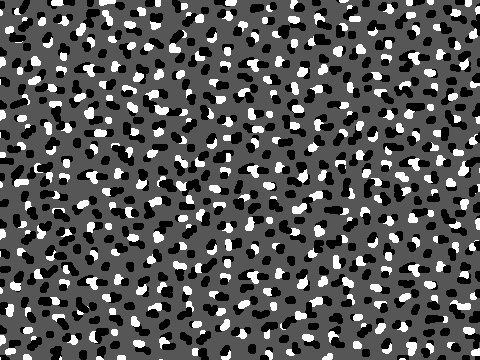


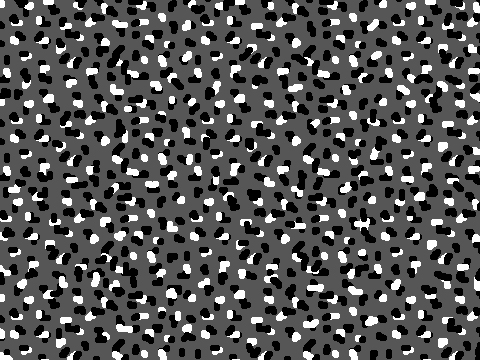

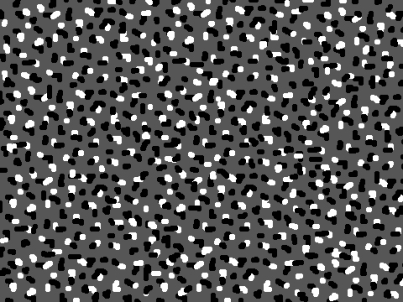

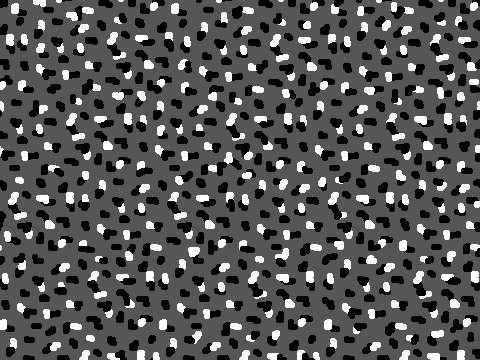


**Figure 1**: The six replicate background types used in Experiments 1 and 2 (not to scale).


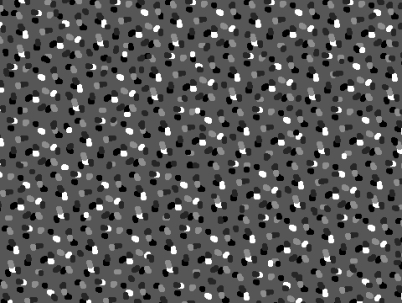

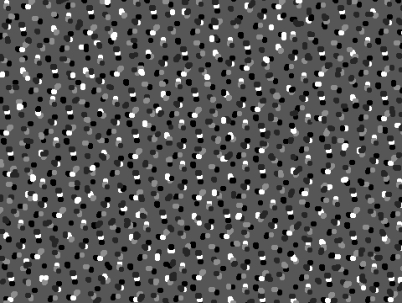

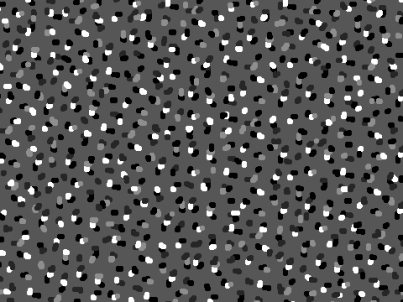


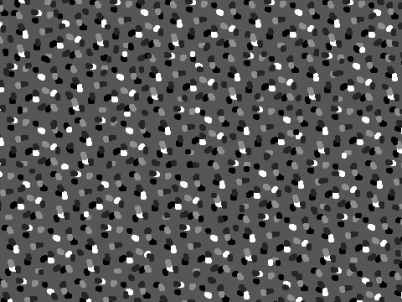

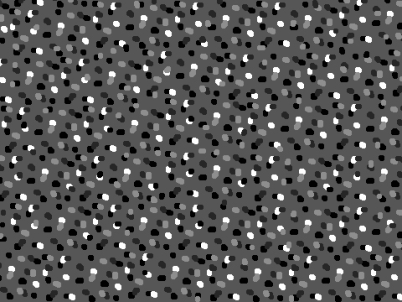

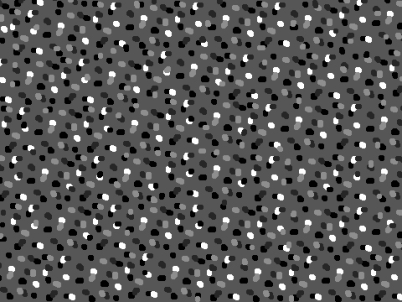


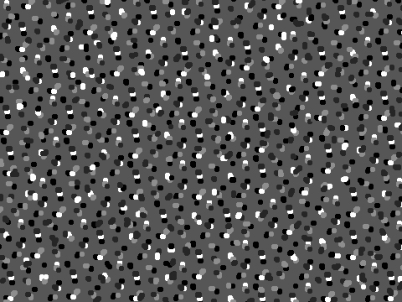

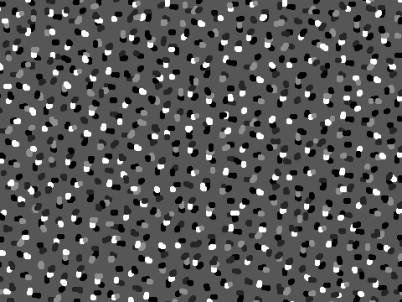


**Figure 2**: The eight replicate background types used in Experiments 3 and 4 (not to scale).
